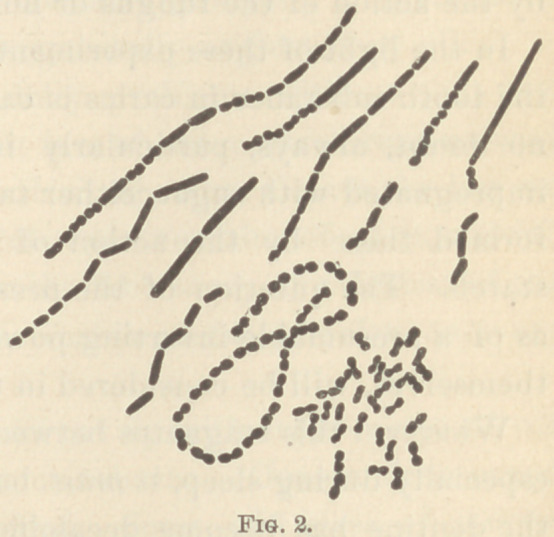# Fermentation in the Human Mouth; Its Relation to Caries of the Teeth

**Published:** 1884-02

**Authors:** W. D. Miller

**Affiliations:** Berlin, Germany


### Independent Practitioner.

VOL. V. FEBRUARY, 1884. No. 2.

### Original Communications.

### FERMENTATION IN THE HUMAN MOUTH; ITS RELATION TO CARIES OF THE TEETH.

BY DR. W. D. MILLER, BERLIN, GERMANY.

During the last two years I have stated at different times and places, as the result of many experiments, that "the first stage of dental caries consists in a decalcification of the tissue of the teeth by acids, which are for the greater part generated in the mouth by fermentation." The object of the investigations described in this and the following papers is to determine this ferment, and the conditions essential to its action. I shall seek in what follows to present no views which are not the legitimate and necessary results of rigid and exact experiment, and I shall give in detail a description of each series of experiments, in order that every one may have an opportunity to judge of the accuracy of the work and the justice of the conclusions drawn from it.

It is, nevertheless, with some hesitancy that I venture to present before the dental profession the results of my last six months' labor, having learned by experience the almost endless number of agents which combine to vitiate such a series of experiments as that which I am about to offer, and the exceeding great care which is necessary in excluding or eliminating all irrelevant factors.

If, therefore, I have been guilty of any oversight, or failed to take all possible precautions to guard against error, I hope that some one will kindly show me where I have gone astray, and put me in the right course again.

The larger apparatus necessary for these experiments are :

1. A large double-walled incubator, with gas regulator for maintaining any desired constant temperature.

2. A Koch sterilizer.

3. A damp chamber. (See Fig. 1.)

4. A drying oven for sterilizing instruments, glass vessels, etc., at a temperature of one hundred and fifty degrees Centigrade.

5. A good microscope, with either water or oil immersion.

Fig. 1.

It is not necessary to mention the smaller instruments, glass vessels, etc., etc., nor the apparatus necessary for making a chemical analysis of the products of the fermentation ; these are sufficiently familiar to every one.

To avoid repetition, I will say here that *all* vessels and instruments used in the culture experiments were purified in the flame of a Bunsen burner, when practicable, otherwise by exposing for fifteen minutes in the drying oven to a temperature of one hundred and fifty to one hundred and sixty degrees Centigrade, (three hundred and two to three hundred and twenty degrees Fahrenheit), and that all substances used as culture substrata were sterilized four times by exposure, at intervals of twelve hours, for half an hour, t osteam at one hundred degrees Centigrade, in a Koch sterilizer. Furthermore, all infections from carious dentine were made as follows : The cavity of a freshly extracted carious tooth is cleared of food, and carefully brushed over with a pledget of cotton dipped in carbolic acid (ninety per cent). The acid is then thoroughly absorbed by means of bibulous paper, and layer after layer of the soft dentine removed with a repeatedly purified instrument, until the deeper parts are reached ; then, a portion of the clean soft dentine, scarcely as large as a pin-head is removed, and quickly brought into or upon the culture medium.

Infections from the mouth were made by scratching upon the surface of the mucous membrane of the cheek, or the margin of the gum, with the end of a clean platinum wire, and then dipping it into the culture medium. The materials used for culture were :

| | | |
|---|---|---|
| *No.* 1. | Sterilized saliva | 50,0 |
| | Sugar | 1,0 |
| | Starch | 0,5 |

*No.* 2. Sterilized milk.

| | | |
|---|---|---|
| *No.* 3. | Decoction of malt | 50,0 |
| | Sugar | 1,0 |

The malt decoction is made by boiling, with slight evaporation, 20,0 dry malt with 120,0 water for ten minutes, and filtering.

| | | |
|---|---|---|
| *No.* 4. | Sterilized saliva | 50,0 |
| | Water | 50,0 |
| | Starch | 20,0 |
| | Sugar | 2,0 |

The starch is added to the cold solution of water and saliva, and stirred until it becomes evenly divided throughout the solution ; it is then poured into shallow glass vessels with glass covers, and put into the sterilizer for complete sterilization ; it there congeals and forms a solid mass, upon the surface of which the infections may be made. It possesses all the advantages of gelatine, with one great additional one, in that it does not liquify at blood temperature.

| | | |
|---|---|---|
| *No.* 5. | Decoction of malt | 100,0 |
| | Sugar | 2,0 |
| | Starch | 20,0 |

Prepared in the same way as No. 4.

| | | |
|---|---|---|
| *No.* 6. | Beef extract | 2,0 |
| | Water | 100,0 |

| | | |
|---|---|---|
| *No.* 7. | Water | 100,0 |
| | Beef extract | 2,0 |
| | Sugar | 2,0 |

*No.* 8. Fresh baked potato, cut into slices one half inch thick, with a clean knife.

Other substances were used, but need not be considered here. Additional sugar is not absolutely necessary where malt is used, though I have, so far, obtained better results by adding a small quantity. The kind of sugar is immaterial, provided it be fermentable; even cane sugar, though not directly fermentable, is converted into a fermentable variety in the culture. Where small quantities of any culture material were used, the cultures were kept in the damp chamber to prevent their drying up or becoming too concentrated by evaporation. All cultures were made under a temperature of thirty-six to thirty-eight degrees Centigrade.

We will begin with the fundamental experiments.

*Exp.* 1. Fresh saliva is mixed with sugar or starch, one to forty, and kept at blood temperature. It invariably becomes acid in four to five hours. But some one, no doubt, will say that this is a result of no consequence, because the experiment was not made within the oral cavity; for his personal benefit we give the following:

*Exp.* 2. A glass tube two c. m. long and three m. m. wide, is filled with starch, sterilized, and fastened to a molar tooth in the mouth on going to bed; next morning the contents of the tube will have a strong acid reaction. A cavity in a tooth, or a piece of linen, which may be saturated with a solution of starch, will answer the purpose as well as the glass tube. That the acid is the same in each case will be further established below.

*Exp.* 3. The mixture of saliva with starch or sugar, is kept for a half-hour in the sterilizer at one hundred degrees Centigrade, and then placed in the incubator; it does not become sour in four, nor in twenty-four hours; in fact not at all. We conclude that the ferment is rendered inactive by a temperature of one hundred degrees Centigrade.

*Exp.* 4. The starch is heated to one hundred and fifty degrees Centigrade before mixing with the saliva; the solution still becomes sour. Conclusion: the ferment exists, not in the starch, but in the saliva.

We have now to determine the question: Is it an organized ferment (fungi,) or is it an unorganized ferment (ptyaline)?

This question is determined by the following experiments:

*Exp.* 5. From six to eight grams of saliva are agitated in a test-tube with as much sulphuric ether as it will take up, starch added, and the whole put in the incubator. On examination after a few hours, we will find sugar in the solution, but no acid; in other words, the acid-forming ferment has been rendered inactive, but the unorganized, sugar-forming ferment, not.

*Exp.* 6. Instead of ether, enough carbolic acid is added to make the solution one-half per cent. strong; the result is the same. These two experiments show that the ptyaline of the saliva (which was not injured by the presence of the ether or the carbolic acid, as proved by the fact that it retained its diastatic action), is not the cause of the acid reaction.

*Exp.* 7. According to Paschutin, ptyaline is devitalized by exposure twenty minutes to a temperature of sixty-seven degrees Centigrade. Organized ferments could not be killed by the same means. We accordingly subject a mixture of saliva and grape-sugar to the given temperature for twenty minutes. We thereby destroy the ptyaline; the mixture, nevertheless, becomes sour if allowed to stand in the incubator for twenty hours.

This experiment confirms the result of experiments five and six, and we begin to suspect that we have to deal with an organized ferment. This supposition is confirmed by the following experiment.

*Exp.* 8. Six to eight drops of a perfectly sterilized solution of sugar in saliva (1-40), in a miniature test tube with cotton cork, are infected from the mouth, or with carious dentine, as described above; in twenty-four hours the solution will be acid; with a fraction of a drop of this solution a second tube is infected; it will likewise becomes acid; from this a third, etc. etc.; each becomes acid in turn, while the control tube (containing the same solution, not infected), remains neutral.

The conclusion is plain, that we have to do with a ferment which is capable of reproducing itself; in other words an organized ferment. It therefore becomes evident that not only free in the mouth, but in the deeper parts of carious dentine, we have a fungus which is capable of producing an acid reaction in characteristic substrata.

*Exp.* 9. Each of thirty small tubes were furnished with eight drops of solution No. 1, and each of thirty other tubes with as many drops of solution No. 3, and all were sterilized. Twenty-four were then infected from the mouth, twenty-four with carious dentine, and twelve were left as controls.

In twenty-four hours all forty-eight of the infected solutions were acid, while the twelve controls remained neutral.

*Exp.* 10. Make a solution of 40,0 of saliva and 1,0 of starch; put equal portions in two flasks, *a* and *b*, and cover the surface of the solution in *a* with a layer of pure oil to prevent the free access of air, or:

*Exp.* 11. Place flask *a* in an air-tight bottle containing a fresh alkaline solution of pyrogallic acid (which abstracts the oxygen from the air), or:

*Exp.* 12. Exhaust flask *a* by means of the air pump, so as to produce a tolerably complete vacuum. The quantity of acid produced in *a*, will be, on an average, the same as that produced in *b*.

We conclude from experiments eight, nine, and ten, that the fungi in question is independent of the free access of air or oxygen for its development and characteristic action, a conclusion which would exclude the fungus of vinegar, (*mycoderma aceti*) and which is of the utmost practical importance, since it signifies that this fungus can develop and perform its work deep in the dentinal tubules, or under fillings, provided the necessary materials are furnished it.

*Exp.* 13. Place a piece of carious dentine upon the surface of the culture material described in number four, five, or six; in twelve hours the dentine will be surrounded by a white ring, from four to eight m. m. in diameter; the material within this ring will be partially liquified, and have an acid reaction. The same result follows when the infection is made from the mouth.

*Exp.* 14. Produce 10,0 of saliva by chewing a sterilized quill toothpick, add 0,5 starch or sugar, and place in the incubator. Then give the oral cavity a most thorough cleansing with pure water, using toothpick, brush and floss, the object being to free the mouth from micro-organisms as completely as possible. Then produce again 10,0 saliva, add 0,5 starch or sugar, and put in the incubator. The amount of acid produced in a given time will, in the latter case, be often as low as one-fourth of that in the former. Conclusion: By thoroughly cleansing the mouth we no doubt remove the greater portion of the fungi, hence the small amount of acid produced. By using strong antiseptics, or by repeatedly filtering the saliva, we may reduce the amount of acid produced in twenty-four hours almost to 0. An experiment yet to be made is to take the saliva direct from the gland, before it becomes infected with the organisms of the mouth; it should not then become sour when mixed with starch and allowed to stand at blood temperature. In every case a careful microscopic examination of the cultures was made, revealing the constant presence of a fungus, chiefly in the form of diplococci, either single or in chains, less often in form of bacteria, bacilli, or even threads. (See fig. 2).

Sometimes all these forms are found on a single thread, thus proving what I have already demonstrated for *Leptothrix buccalis* and *Leptothrix gigantea* (Miller), the genetic connection of these different forms. The particular form in which the fungus occurs depends somewhat upon the culture medium, as well as upon the age of the culture. By using a glass tube as culture vessel we may demonstrate that whether the culture is made in the mouth or out of it, under similar conditions the fungus is the same. The fungus is not capable of producing an acid reaction of all substances in which it may vegetate. A luxuriant growth may be obtained in beef extract, but no acid is produced, unless sugar is present.

FIG. 2.

It is only from carbo-hydrates (especially sugar) that it appears to be able to produce acid in any considerable quantity, or at all. This question, however, as well as the morphology, physiology, development and life-conditions of the fungus, will receive consideration in a separate number.

We have, then, a micro-organism which agrees morphologically with the *Bacterium acidi lactici*, and which, without the presence of oxygen, produces acid from sugar, so that we would probably not be far from right if we were to say that the organism in question is simply the fungus of lactic acid; we will, however, reserve our decision for the following number, where the analysis of the product of the fermentation will be given, that being the one *sure* method for determining the species of any ferment bacterium.

In all cultures, it is of course essential that the culture-substratum be neutral when the inoculation is made; should it be acid it must be neutralized. This is best accomplished by very carefully adding the carbonate of sodium. Without this precaution it would be somewhat difficult to determine whether acid had been produced by the action of the fungus or not.

In the light of these experiments the thorough decalcification of the tooth substance in caries is easily accounted for. The saliva is, no doubt, always, particularly in mouths of uncleanly persons, impregnated with sugar, either taken directly into the mouth, or formed there by the action of the ptyaline of the saliva upon starch. The question of the presumable diastatic action, as well as of a presumable inverting power on the part of the organisms themselves, will be considered in the chapter on Physiology.

Wherever this stagnates between the teeth, in fissures, etc., etc., especially during sleep, it *must* become acid. When a portion of the dentine has become decalcified, it, as is well known, takes up the liquids of the mouth and the fungi with them like a sponge, and the fungi, being independent of the free access of air, go on producing acid within the dentinal tubules. As each layer of dentine becomes softened in turn, the micro-organisms *follow after*, continually producing new acid. Hereby the zone of softened, non-infected dentine, is readily understood. The production of acid is entirely independent of the reaction of the saliva as it enters the mouth, hence the uselessness of "testing the saliva" for acid. That the liquid squeezed out of the tubules of *decaying* dentine has an acid reaction, every dentist in America who has a piece of blue litmus paper and is not color blind, can easily prove for himself.

The result of experiment Six plainly shows one cause of the good effects which the profession has seen from the use of carbolic acid.

The fact that a pure culture was obtained in most cases by the first inoculation, seems to indicate that the fungus exists in a state of tolerable purity in the deeper parts of the carious dentine. This question will, however, receive consideration later. The action of the fungus upon substances which contain no carbohydrates will also be considered under Physiology.

## Figures and Tables

**Fig. 1. f1:**
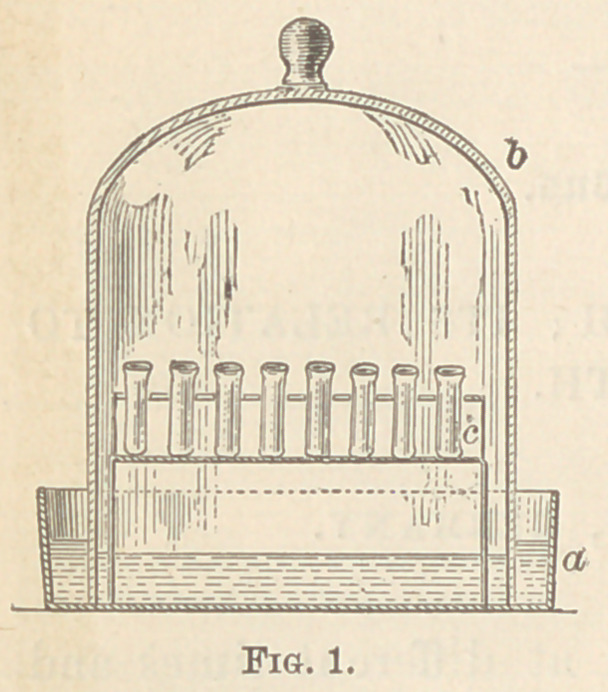


**Fig. 2. f2:**